# Analysis of Pleiotropic Transcriptional Profiles: A Case Study of DNA Gyrase Inhibition

**DOI:** 10.1371/journal.pgen.0020152

**Published:** 2006-09-29

**Authors:** Kyeong Soo Jeong, Yang Xie, Hiroshi Hiasa, Arkady B Khodursky

**Affiliations:** 1 Department of Biochemistry, Molecular Biology, and Biophysics, University of Minnesota, St. Paul, Minnesota, United States of America; 2 Biotechnology Institute, University of Minnesota, St. Paul, Minnesota, United States of America; 3 Division of Biostatistics, School of Public Health, University of Minnesota, Minneapolis, Minnesota, United States of America; 4 Department of Pharmacology, University of Minnesota Medical School, Minneapolis, Minnesota, United States of America; North Carolina State University, United States of America

## Abstract

Genetic and environmental perturbations often result in complex transcriptional responses involving multiple genes and regulons. In order to understand the nature of a response, one has to account for the contribution of the downstream effects to the formation of a response. Such analysis can be carried out within a statistical framework in which the individual effects are independently collected and then combined within a linear model. Here, we modeled the contribution of DNA replication, supercoiling, and repair to the transcriptional response of inhibition of the Escherichia coli gyrase. By representing the gyrase inhibition as a true pleiotropic phenomenon, we were able to demonstrate that: (1) DNA replication is required for the formation of spatial transcriptional domains; (2) the transcriptional response to the gyrase inhibition is coordinated between at least two modules involved in DNA maintenance, relaxation and damage response; (3) the genes whose transcriptional response to the gyrase inhibition does not depend on the main relaxation activity of the cell can be classified on the basis of a GC excess in their upstream and coding sequences; and (4) relaxation by topoisomerase I dominates the transcriptional response, followed by the effects of replication and RecA. We functionally tested the effect of the interaction between relaxation and repair activities, and found support for the model derived from the microarray data. We conclude that modeling compound transcriptional profiles as a combination of downstream transcriptional effects allows for a more realistic, accurate, and meaningful representation of the transcriptional activity of a genome.

## Synopsis

Pleiotropism—a movement, or reaction, in multiple directions: although it was initially used specifically to describe the effect of a single genetic mutation on multiple characters in the offspring, the transcriptional responses of cells are often best described in terms of pleiotropy, when a single input affects multiple components inside the cell. This, in turn, presents a dilemma with the analysis and interpretation of the observed effects: which effects are directly due to the input itself and which are not? How are the effects related to each other and which are more important? And finally, can the overall transcriptional response be summarized as a combination of the effects? There is, however, a problem with recording the effects when they occur almost simultaneously in the same organism. The authors approached this by recording the effects independently, using mutants that could generate all of the effects of interest but one, and then estimating the effects and their interactions from a multivariate linear model. The authors applied this method to explain the transcriptional response of Escherichia coli to a quinolone antibacterial, a relative of Cipro (ciprofloxacin hydrochloride), and discovered unexpected interactions between DNA maintenance modules in the cell.

## Introduction

DNA gyrase is an enzyme ubiquitously present throughout the bacterial kingdom, with a central role in DNA maintenance and chromosome metabolism in the cell: it is essential for initiation and elongation of DNA replication, and for chromosome segregation [1,2]. These cellular processes are dependent on the supercoiling activity of gyrase. Inhibition of that activity by genetic or pharmacological means disrupts these processes and may cause irreversible DNA damage leading to bacterial cell death [3]. Before the advent of genomics tools, the consequences of gyrase inhibition could be evaluated on three levels: (1) global effects on growth, replication, transcription, and translation; (2) local effects on transcription of selected genes; and (3) biochemical effects on plasmid supercoiling.

All these studies, while implicitly acknowledging the pleiotropic nature of the gyrase inhibition, could not adequately address or incorporate the pleiotropicity into the analysis, given the state of technology at the time. The ability to monitor transcriptional activity of entire genomes allowed an assessment of transcriptional and replication states of the Escherichia coli chromosome following inhibition of DNA gyrase [4–6]. These studies confirmed, now on a genome-wide scale, that treating cells with the gyrase inhibitors affects transcription of a large number of genes in the genome and arrests the movement of the replication fork(s). A systematic analysis of transcriptional effects would not be possible, however, without accounting for a multiplicity of cellular responses triggered by the gyrase inhibition. Such known responses include DNA damage, DNA relaxation, and arrest of replication [4,7–9]. It is critical to be able to model the transcriptional response accounting for those effects because the loss of the gyrase activity radiates through the system as a function of at least that many variables. Such a formulation underscores the fact that observed transcriptional effects almost never can be adequately interpreted in terms of one factor, regardless of how convenient it may seem. Numerous conditional and genetic changes inflicted upon biological systems result in complex responses whose nature cannot be properly elucidated without evaluating the contribution of affected processes and regulatory modules. The failure to do so may result in an incomplete or even an erroneous interpretation of data.

In the reported study, we constructed isogenic mutant stains that were deficient in generating each one of the known cellular responses to the inhibition of DNA gyrase. We collected transcriptional responses associated with the loss of the gyrase function in those strains, and used the collected parameters to model and interpret the DNA gyrase–dependent global patterns of transcriptional activity in the E. coli genome.

## Results

### Inhibition of DNA Gyrase Affects Transcription Globally and Locally

Genome-wide transcriptional profiles have been widely and successfully used to comparatively describe physiological states of various organisms. Most treatments and genetic perturbations, however, result in complex transcriptional responses spreading over multiple regulatory pathways, functional groups, and physiological processes in a cell. Thus, in order to adequately evaluate a transcriptional outcome, such as a resulting transcriptional profile of a treatment, one has to assess the contribution of individual effects (pathways, groups, and processes) to the formation of a response and the interactions between the effects. We chose to dissect the compound response of gyrase inhibition for the following reasons: (1) physiological and transcriptional data indicate that multiple processes are affected by the loss of gyrase function; (2) the main affected processes can be genetically manipulated and excluded from the contribution to the profile; (3) targeting DNA gyrase, an essential bacterial enzyme, is an effective therapeutic means of killing pathogenic bacteria, and (4) the cellular and environmental requirements for efficient targeting of gyrase are insufficiently understood. In the study, we used a prototypical fluoroquinolone, norfloxacin, to inhibit the gyrase activity. The experiment was designed as a time-course treatment at a drug concentration that was effective against wild-type cells and not against a mutant stain carrying a drug-resistant gyrase allele. First, we established the overall nature of the transcriptional response triggered in the bacteria by targeting gyrase in the defined growth conditions. Relative transcript abundances were determined at 5, 10, 15, and 20 min after the addition of norfloxacin. Using measurements from four different time points, we concluded that the transcriptional activity of about 15% of the bacterial genome (613 genes) is affected by the targeting DNA gyrase (false discovery rate [10] is less than 1%). To ensure that this transcriptional response was specifically mediated by the inhibition of DNA gyrase, we examined the effect of the drug on genome-wide transcription in a mutant carrying a quinolone-resistant allele of *gyrA, gyrA* ^r^ *;* S83L [11]. We found that the norfloxacin treatment of the resistant mutant caused no changes in transcription, with the exception of up-regulation of the genes in the ArgR regulon, the effect opposite to the one observed in the wild-type cells. Overall, the inhibition of gyrase led to up-regulation of 269 and down-regulation of 344 genes. The affected genes belonged to various functional categories dominated by those whose products maintain DNA integrity and mediate transport and diffusion of low-molecular-weight compounds (Table S1). The response can be rationalized as a bacterial defense strategy to reduce intake of the drug, repair DNA damage, and restore DNA conformation and regulation in the cell. In agreement with the earlier reports, the targeting of DNA gyrase resulted in induction of the SOS regulon [12], up-regulation of the *gyrA* and *gyrB* genes [13], and repression of the *topA* gene [6].

**Figure 1:**
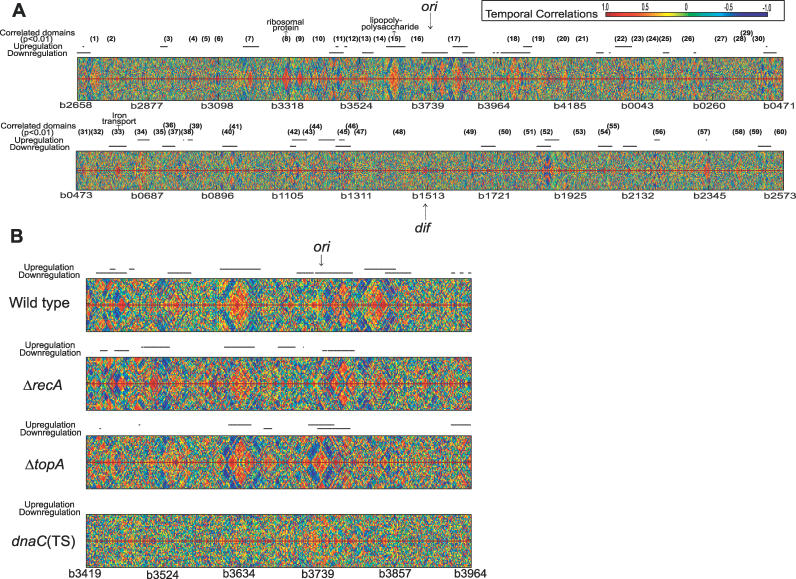
Spatial Correlations of Temporal Transcriptional Profiles Elicited by Gyrase Inhibition Temporal transcriptional profiles observed in the wild-type (A) and isogenic mutant strains (B). Pair-wise correlations of temporal transcript profiles of 4,000 genes, arranged in the chromosomal order, are shown on a colorimetric scale for the whole chromosome (A) and the *oriC* proximal region (B). For spatial correlations around the *n*-th gene (*n* on the *x*-axis) in the chromosomal order, each *y*-axis includes 20 Pearson correlations of temporal profile pairs, (*n* − 1, *n* + 1), (*n* − 2, *n* +2),...(*n* − 19, *n* + 19), and (*n* − 20, *n* + 20). Chromosomal regions with high correlations exhibit symmetric red (positive) or blue (negative) triangles. Differentially expressed genes were significantly enriched in the regions marked with horizontal bars. The regions in which at least 16 consecutive genes show significant high correlations are enumerated. The statistical significance is estimated by the comparison with 5,000 randomly sampled subsets of temporal profiles.

We also examined the profile from a different angle: for existence of spatial transcriptional domains. Such analysis provides important regulatory and organizational information, which is invariably missed when the location of genes on the chromosome is not accounted for [14]. We observed the formation of local transcriptional domains [15] as a result of the gyrase inhibition (Figure 1). Spatial correlations of temporal profiles (0–20-min time series) suggested that some sets of genes linearly juxtaposed on the chromosome underwent similar transcriptional changes as a result of the treatment. We identified 60 domains larger than 3 kilobases (kb). (In Figure 1, domains were delineated using a sliding window of 16 consecutive genes, the expected length of the short-range transcriptional patterns [14].) Twenty-five of those domains overlapped with the regions enriched for differentially expressed genes. Genes in several domains belonged to well-defined functional classes, such as: ribosomal proteins, domain 8; lipopolysaccharide synthesis, domain 15; and iron transport proteins, domain 33. Domain 15 spanned across 32 genes, in a region from *b3616* to *b3650*. This region contains two operons of the lipopolysaccharide synthesis genes *(rfa* genes: *b3619*~*b3632)* along with some functionally unrelated open reading frames (ORFs). Differential and coherent regulation of lipopolysaccharide synthesis may contribute to bacterial defense mechanisms, which would be consistent with the earlier observation linking bacterial resistance to norfloxacin with the levels of lipopolysaccharide in the cell [16,17]. Interestingly, despite the uniform distribution of differentially affected regions along the chromosome, the transcriptional domains were very sparse in the region surrounding the terminus of replication. This observation was consistent with our earlier finding that the spatial correlations of transcriptional activity in this region of the chromosome were particularly sensitive to the gyrase activity [14].

### Uncoupling the Effects of Relaxation, Repair, and Replication

Inhibition of the supercoiling activity of DNA gyrase in vivo is confounded by at least two other global effects, inhibition of DNA replication and DNA breaking. We were not able to find a condition, including a drug treatment or a temperature-sensitive mutation, in which inhibition of plasmid supercoiling would not be accompanied by induction of the prototypical SOS response and by an arrest, or slowing, of DNA replication (unpublished data). Moreover, the transcriptional effects of inhibition of gyrase are assumed to result from relaxation/overwinding of DNA, but it has not been demonstrated that the observed effects are indeed dependent on a relaxation activity of the cell. To untangle these effects, we examined the transcriptional response in the mutants in which the transcriptional effect of gyrase inhibition was not interfered with by the DNA damage response or replication arrest. We also examined transcriptional consequences of the gyrase inhibition in a strain lacking the major relaxation activity, topoisomerase I (Topo I). To that end, we constructed three isogenic strains in the MG1655 genetic background: a first mutant carried a deletion of the *recA* gene and could not generate the SOS response; a second, a *dnaC* temperature-sensitive mutation, could not initiate DNA replication at 38 °C; and a third, a deletion of the *topA* gene, could not efficiently relax negatively supercoiled DNA. Treatment of these strains with norfloxacin produced markedly different results. As expected, the *recA* deletion made the cells on average more susceptible to the drug: only 2% of the Δ*recA* cells survived a 20-min treatment compared to 25% of the wild type (see Table S2). Unexpectedly, the Δ*topA* strain showed a slightly higher viability than the wild type. And even more surprisingly, the *dnaC*(Ts) strain was even more susceptible to the drug in the absence of ongoing replication than the wild type (12% of cells survived after 20 min; Table S2). Cytotoxicity of quinolones has been assumed to be triggered when a DNA replication fork collides with a topoisomerase-DNA-quinolone ternary complex [9,18,19]. Significant cell killing in the *dnaC*(Ts) mutant indicated that the passage of the DNA replication fork is not necessary for the drug cytotoxicity.

In addition to the different drug sensitivities, these strains demonstrated diverse patterns of a local transcriptional activity. Although individual strains could be characterized by some unique spatial domains, many of the domains could be seen in three out of four strains, including most prominently the domain surrounding the *rfa* locus (Figure 1B). Moreover, spatial transcriptional domains could not be detected in the absence of DNA replication, even though the cells were fully transcriptionally competent (based on an isotope incorporation study and *lac*-operon induction; unpublished data).

### Comparative Overview of the Individual Strains

The Δ*recA* strain, in addition to the loss of the recombination function, is deficient in a RecA-mediated cleavage of the LexA protein that represses transcription of the SOS genes [12]. Following the drug treatment, all of the known genes in the LexA regulon were activated in the RecA-dependent manner (Table 1). The regulatory regions of the genes that showed RecA-dependent activation contained the LexA binding site more frequently than expected by chance (Table 1; CTG[N10]CAG, *p* = 2.3E−13; TTG[N10]CAG for *dinG, p* = 4.4E−3, assuming a hypergeometric distribution for testing against the null hypothesis). Some other genes containing the LexA box, including *argS, cydD, glyA, gor, rfaZ, trxA, yccS, ychB,* and *yecD,* also exhibited RecA-dependent activation. Although the involvement of some of those genes in the SOS response has been sporadically addressed (for example, it has been shown that a thioredoxin-deficient mutant *(trxA)* is sensitive to hydrogen peroxide [20]), their role in rescuing bacterial cells from DNA damage remains unclear.

**Table 1:**
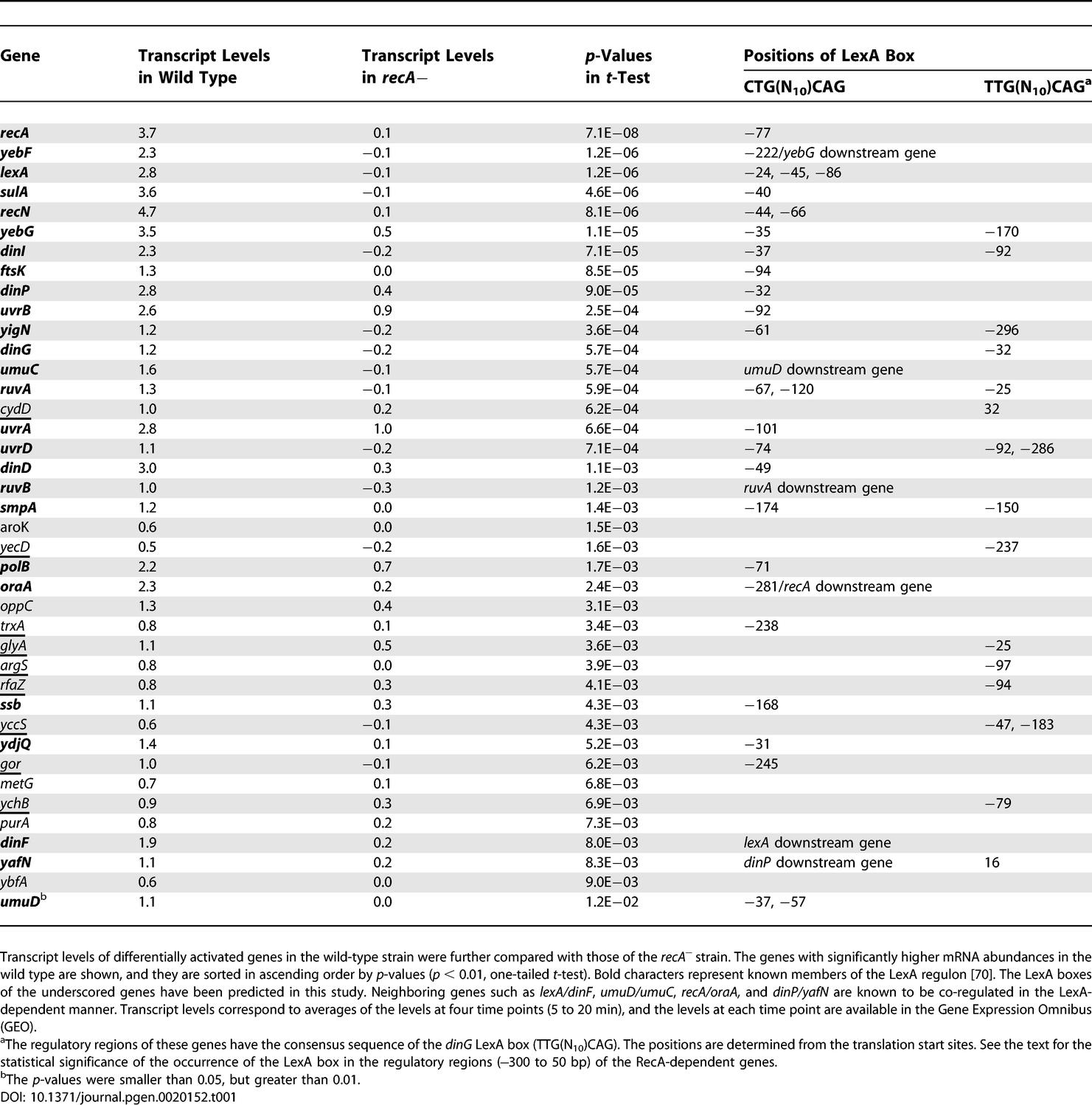
RecA-Dependent Activation and Genes of the LexA Regulon

The E. coli strain carrying the deletion of the *topA* allele without the compensatory mutations has been reported recently [21]. We confirmed that transduction of the Δ*topA* into the MG1655 background was not accompanied by the compensatory mutations in the *gyrA* or *gyrB* genes or by any changes in the *tolC-par* and *topB* regions of the chromosome (unpublished data). We also did not observe any changes in transcript or protein levels of the *parC, parE,* and *topB* (unpublished data). Additionally, we verified that a significant fraction of the plasmid DNA was hyper-negatively supercoiled in the Δ*topA* strain and that positively supercoiled plasmid DNA did not accumulate in the strain even at 50 μg/ml of norfloxacin; only traces of the relaxation activity could be observed at 37 °C, but not at 30 °C, when DNA gyrase was inhibited by the drug (Figure S1). The direct comparison of the transcriptional profiles between the Δ*topA* strain and its isogenic wild type without the drug treatment revealed that the most up-regulated genes in the Δ*topA* strain were the genes in the *leuABCD*/*leuO* cluster (*b0071*~*b0076,* 2.6- to 4.8-fold induction). This activation of the *leu* gene cluster is reminiscent of the suppression of the *leu*-500 promoter by the *topA* mutation in Salmonella typhimurium [22,23]. In *E. coli,* it has been demonstrated that the plasmid-borne *leu*-500 promoter can be activated by the *topA* mutation when the diffusion of free supercoils is presumably constrained [24].

Besides the *leu* cluster, genes in several other functional groups appeared to be activated in the absence of the *topA,* including energy metabolism (*acnB, aldA, gltA, fruR, fumC, lldD,* and *pdhR;* at least 2-fold change), transport and binding proteins *(fadL, lamB, lldP, malE,* and *rbsD),* and central intermediary metabolism *(metK, pps,* and *speB)*. The majority of the down-regulated genes belonged to the “cell envelope” (22 genes) and the “motility and chemotaxis” (ten genes) categories.

At a restrictive temperature of 38 °C, the *dnaC*(Ts) mutation blocks the initiation of DNA replication while allowing ongoing rounds to go to completion [25,26]. Shifting the *dnaC*(Ts) strain and its isogenic wild type from 30 °C to 38 °C resulted in the indistinguishable transcriptional profiles (G. Prasad, K. S. Jeong, and A. B. Khodursky, unpublished data). After the completion of ongoing rounds of replication, 90 min after the upshift, the cells were treated with norfloxacin, and the transcriptional response was evaluated relative to the untreated control. Although the SOS response was subdued compared to the response in the wild type, it could be easily discerned from the global transcriptional profile (Figure S2). We also observed up-regulation of the genes encoding DNA gyrase, *gyrA* (~1.6-fold), and *gyrB* (~2.5), and down-regulation of the *topA* (0.5), which was similar to the wild-type response.

### Multifactorial Analysis of the Gyrase-Dependent Transcriptional Response

**Figure 2:**
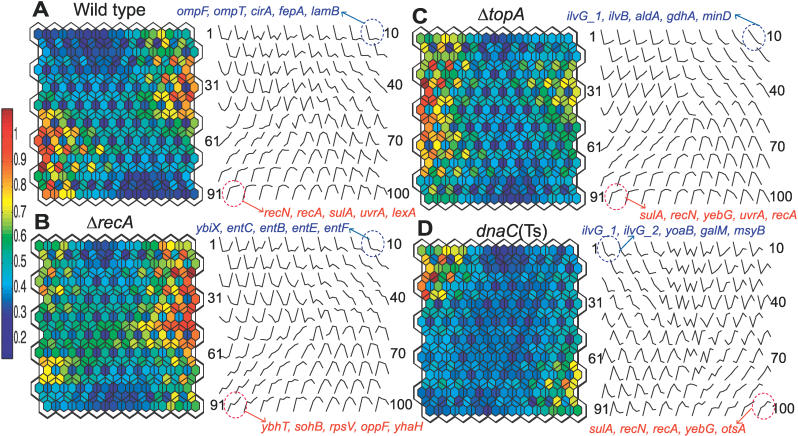
SOM Analysis of Temporal Transcriptional Profiles U-matrix (left side of panel) and weight vectors (right side of panel) of a self-organizing feature map of temporal transcriptional profiles observed after the addition of norfloxacin to the wild-type (A), *recA* (B), *topA* (C), or *dnaC*(Ts) (D) mutant. Transcript levels measured at 5, 10, 15, and 20 min of the treatment were compared with those of the non-treated cells (0 min; first time point) for 1,109 genes that showed differential expression in at least one strain. Each contoured hexagon (left side of panel) has a corresponding weight vector that represents temporal responses (0 to 20 min, right side of panel). Small hexagons indicate map units that are colored according to the medians of the surrounding hexagon with a contour line passing through.

The cluster analysis of the time-course data using self-organizing maps (SOM) [27] revealed substantial differences in the temporal expression patterns among the four strains (Figure 2). The expression patterns shown by the various weight vectors of the SOM indicated that the coordination of the transcriptional response in the cells is affected by the individual mutations. For example, although in the wild-type cells the activity of the SOS regulon is most anti-correlated with the activity of the major transporter systems (Figure 2A, the bottom-left corner and top-right corner vectors, circled), this relationship is broken in all three mutants. Whereas this is not particularly surprising in the case with the *recA^−^* strain in which the SOS genes were not induced (Figure 2B; no specific nodes could be identified with any subset of the SOS genes), it is rather unexpected that, in the absence of the *topA* gene, the SOS genes should be most anti-correlated with the different group of genes, those involved in the cell division and amino acid metabolism (Figure 2C). Additionally, whereas some differentially expressed genes exhibited similar temporal profiles in different strains (red or blue circle), a gene composition of each profile was strongly affected by a mutant background. These variations, however, cannot be adequately accounted for by an exploratory data analysis, such as SOM.

To explain these variations and to obtain a synthetic description of the transcriptional consequences of the gyrase inhibition in the wild-type cells, we developed a linear model that took into account the multiplicity of physiological effects stemming from the gyrase inhibition (see Materials and Methods). The model incorporated seven effects: the effect of norfloxacin on the wild-type cells (coefficient *β**_1_***); the genetic effects associated with the ability of the cells to repair DNA (coefficient *β**_2_***), to relax DNA *(β**_3_**)*, and to replicate DNA *(β**_4_**)*; and the effect of norfloxacin on the cells that cannot repair their DNA *(β**_5_**)*, cannot efficiently relax their DNA *(β**_6_**)*, and do not replicate their DNA *(β**_7_**)*. For example, the coefficients *β**_1_**, β**_5_**, β**_6_**,* and *β**_7_*** characterizing the activity of one of the SOS response genes, *sulA,* are, respectively, 3.6, −3.7, −1.2, and −2.3. It signifies that the transcriptional induction of *sulA* in the wild-type cells by norfloxacin *(β_1_)* is negated in the *recA*-deletion mutant (drug–*recA* interaction: *β_5_*), with the two effects acting in the opposite directions (3.6 versus −3.7). Besides the effect of the *recA* mutation, the *topA* and *dnaC* mutations also influenced the induction of the *sulA* gene, although to a somewhat lesser extent. The compilation of all coefficients determined for the genes present on the DNA microarrays is provided in Table S3.

**Figure 3:**
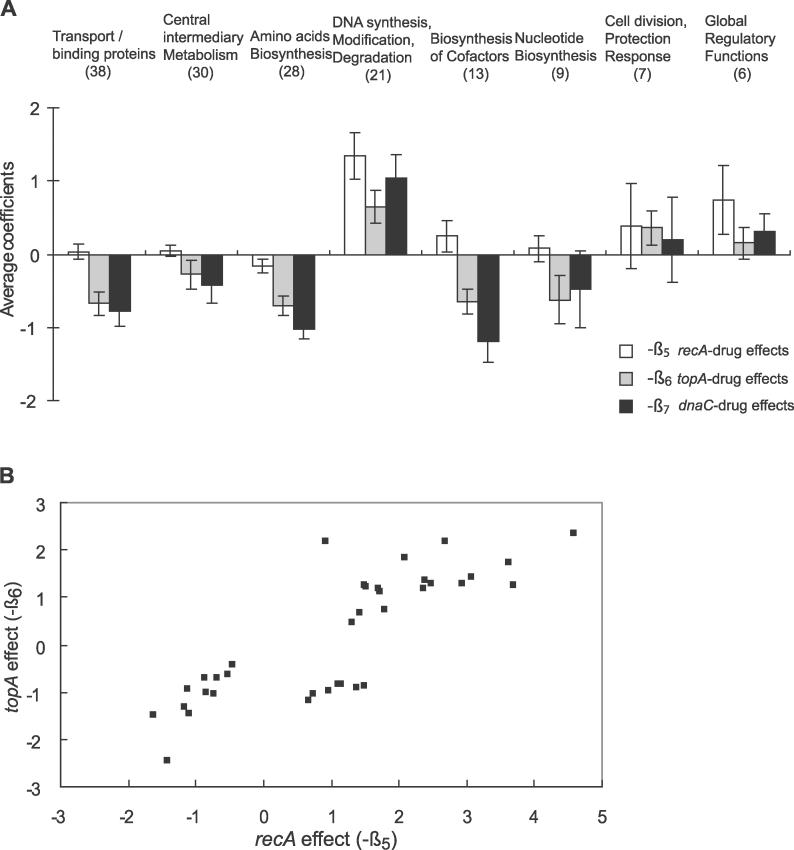
Average Coefficients of Gene–Drug Interactions The *β**_5_**, β**_6_**,* and *β**_7_*** values in different functional groups (A) and association between the *recA* and *topA* mutation effects (B) are shown. (A) For the sake of convenience, the average coefficients are shown with opposite signs (−β*_5_* *,* −β*_6_*, and −β*_7_*), so that the positive values indicate the increase in transcript levels and the negative indicates the decrease. The number of genes in each functional group is in parenthesis, and the error bars represent two standard errors. (B) The interactions between the *recA–*drug *(−β_5_*) and *topA–*drug *(−β_6_*) effects can be seen as the positive correlations for 36 selected genes *(r* = 0.82; *p* < 0.1 in both −β*_5_* and −β*_6_*).

We observed that at least one coefficient, a ratio in a typical context of pair-wise comparisons, describing the gene–drug interactions changed by at least 2-fold (*β**_5_**, β**_6_**,* or *β**_7_*** > 1 or < −1) for 230 genes representing eight functional categories (Figure 3A). For example, the genes whose products are annotated as related to transport/binding proteins were repressed in the presence of the functional *topA* or *dnaC* gene (In Figure 3, the positive values represent a relative increase in transcript levels in the presence of a wild-type allele and vice versa). Transcription of the genes encoding components of central intermediary metabolism, amino acid biosynthesis, and biosynthesis of cofactors has been similarly affected. The *recA* gene contributed mostly to activation of the genes in the DNA synthesis, modification, and degradation category. In addition to the *recA*–drug effect *(β**_5_**)*, the *topA*–drug *(β**_6_**)* and *dnaC*–drug *(β**_7_**)* interactions suggested that the loss of function of the *topA* or *dnaC* gene can substantially affect transcriptional activation of those genes. In this group, the coefficients *(−β**_5_**)* for the *recA*–drug interaction are larger than those *(−β**_6_**)* for the *topA*–drug interaction (*p* = 0.03), but they are comparable to those *(−β**_7_**)* describing the *dnaC*–drug interaction (*p* = 0.25). For most genes with known functions, the transcriptional response to the gyrase inhibition appeared to be associated predominantly with DNA relaxation *(−β**_6_**)* and/or DNA replication *(−β**_7_**)*, with the *recA* contributing mostly to transcriptional activity of the genes involved in DNA metabolism and cell division.

### A Role of Topo I in Mediating a Relaxation-Dependent Transcriptional Response

**Figure 4:**
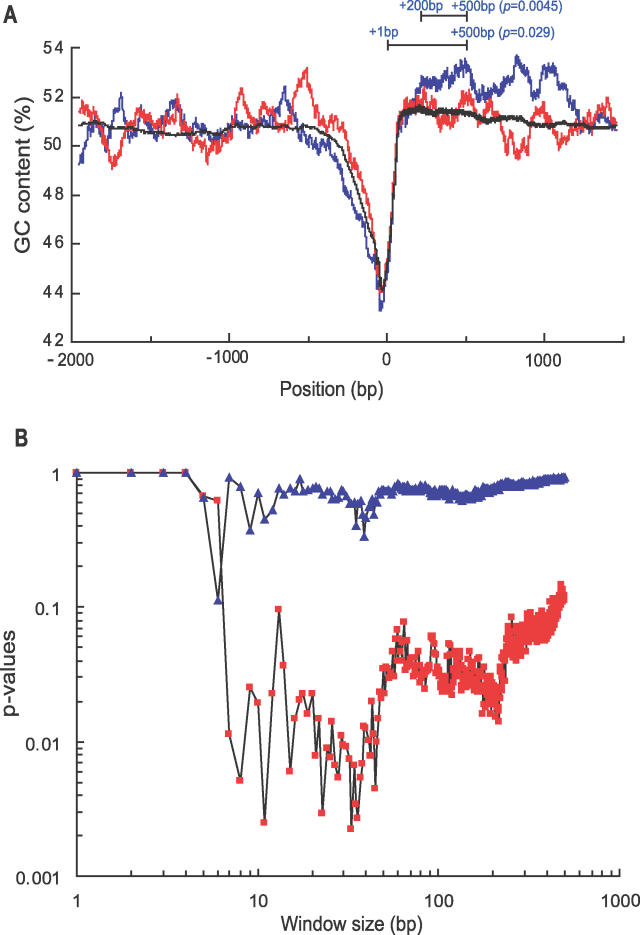
Comparison of the GC Content in Upstream and Coding Regions of Activated and Repressed Genes (A) The GC content of the DNA regions from −2,000 to +1,500 nt, relative to the translation start site, of 84 activated (red) and 95 repressed (blue) genes was calculated with a 100-nt moving window (black: the average GC content of the genome). The significance of the GC content differences in a given region was determined by comparing the average GC content of the repressed genes with the GC content of 10,000 randomly sampled sets of similar fragments in the genome. (B) DNA windows, from 1 nt to 500 nt, were compared to assess their GC content in −500-nt upstream regions. The *p*-values were determined by comparing the averages of the maximal GC content values of the activated (red square), or repressed (blue triangle), genes with the corresponding values of 10,000 randomly sampled sets from the whole genome.

Transcriptional effects of DNA gyrase inhibition, repression, or activation of transcription [5], are assumed to be associated with DNA relaxation. Topo I is the main relaxation activity in the cell [21]. To examine the contribution of Topo I to the spectrum of transcriptional events triggered by gyrase inhibition, we identified the set of genes that were differentially affected by the drug, compared to those without using the drug, in the wild-type strain (*q* < 1% in SAM [significance analysis of microarrays] and coefficient *β**_1_*** > 0.5 or < −0.5). These genes can be further classified into four groups according to their *topA* dependency (Table 2) as follows: group I: activated, *topA* independent; group II: activated, *topA* dependent; group III: repressed, *topA* independent; and group IV: repressed, *topA* dependent. The inhibition of DNA gyrase by norfloxacin activated 84 genes (group I) and repressed 95 genes (group III), in both the wild type and Δ*topA* mutant. Regulation of transcription of those genes depended on changes in the gyrase activity, but not on the activity of Topo I. Interestingly, the repressed genes had a relatively high GC content in their coding regions, uncharacteristic of the E. coli genome sequence, (group III, blue in Figure 4A; *p* = 0.029, coding regions between +1 and +500 base pairs [bp] from the translation start sites; *p* = 0.0045 between +200 and +500 bp), whereas the activated genes had the expected GC content (group I, red in Figure 4A). Since their regulatory regions had a slightly lower GC content than expected, repression of those genes upon the gyrase inhibition could not be explained on the basis of an unfavorable energetic state of the DNA template on the stage of transcription initiation. Instead, it is more likely that efficient elongation of transcription through the regions with the high GC content requires a swivel, normally provided by DNA gyrase.

**Table 2:**
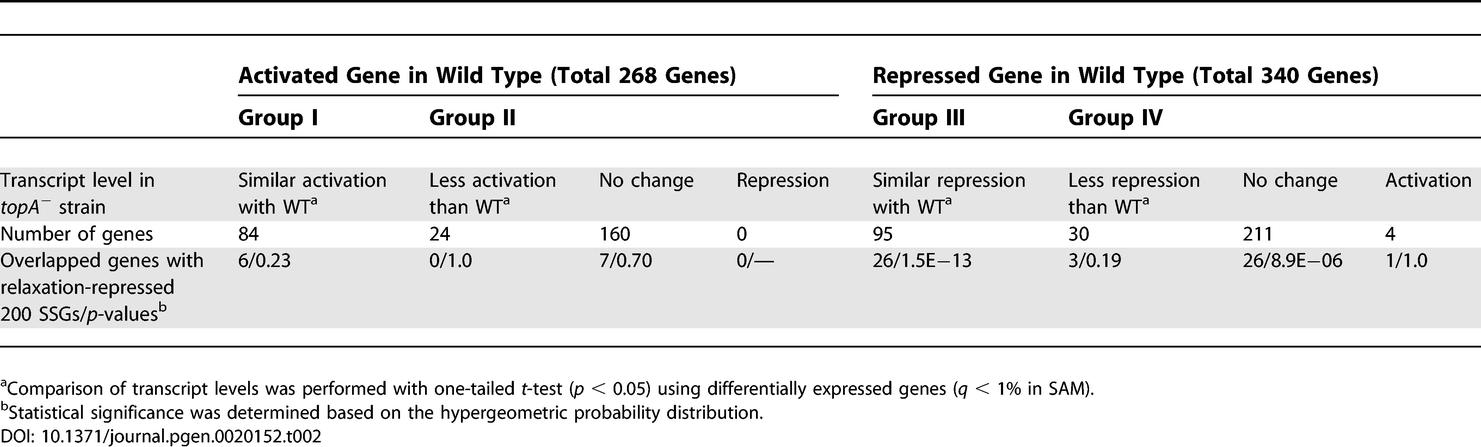
A *topA*-Dependent Classification of Differentially Expressed Genes

Unlike the genes that were repressed by inhibition of gyrase, the upstream regions (up to about 1 kb from their respective translation start sites) of the activated genes had a biased GC composition (data series labeled in red in Figure 4A). We found that short DNA segments with an unusually high GC content are located in the upstream regions of the activated genes (group I in Table 2; red in Figure 4B). When compared with 10,000 randomly selected sets of sequences from the entire genome, the windows with the most significant differences in the GC content were 33, 11, 36, 23, and 35 bp (*p* = 0.0022 to 0.0034). These sizes are very similar to those of palindromic units (PU or REP; sizes range from 33 to 40 bp [28,29]). Several PUs can be found in the bacterial interspersed mosaic elements (BIMEs) that are known to be preferentially bound and cleaved by DNA gyrase [29–31]. The PUs are imperfect palindromes, and their partial DNA base composition exhibits an unusually high GC content (Y: 13 bp, 85%; Z1: 9 bp, 78%; and Z2: 14 bp, 86%). We found several significant GC-rich consensus sequences such as CGCGCCG (*p* = 6.5E−14) and GCGGCGCGC (*p* = 6.4E−7) in 500-bp upstream regions of 84 activated genes using Bioprospector [32]. The sizes of the DNA fragments with a high GC content found in those regions ranged from 8 to 46 bp (*p* < 0.01). Such GC-rich motifs were not found in the sequences of the repressed genes (group III, blue in Figure 4B). It is tempting to speculate that these small GC-rich sequences, similar to BIMEs, could serve as mediators of the gyrase activity on the chromosome. Interestingly, the majority of the gyrase binding sites mapped by Franco and Drlica [33] were located in the GC rich region of the *recF* gene.

Many genes, whose transcriptional activity was repressed by gyrase inhibition in the wild type, exhibited no, or insignificant, changes in transcript levels in the Δ*topA* strain (group IV: 245 genes, 72%; see Table 2). It is very likely that the normal transcriptional activity of those genes requires normal levels of DNA supercoiling, and they become repressed when the DNA template is relaxed by Topo I. The following classes were significantly represented by the genes from group IV: transport/binding proteins (47 genes), amino acid biosynthesis (32), macromolecule synthesis (17), central intermediary metabolism (19), and nucleotide biosynthesis (11). As expected [6,34], this group included the genes whose transcriptional activity is known to be sensitive to the superhelicity of DNA, such as *ompF*, *lamB,* and *proVWX*.

### The Interaction between RecA and Topo I

Although our experimental design did not allow the direct quantification of the interactions between the genetic factors, we explored possible associations by a pair-wise comparison of the relevant effects. Because of the apparent role of DNA replication in establishing the drug-related transcriptional response (see above) and the overall correlation between the *recA* and *topA* effects (*r* = 0.3 for 4,069 genes), we focused only on examining the relationship between the effects of *recA* and *topA* on the drug-induced transcriptional changes. From our linear model, we identified 36 genes whose expression appeared to be affected by the drug in the *recA*- and *topA*-dependent manner (see Figure 3B). For this group of genes, we observed a strong correlation (*r* > 0.8) between the changes in transcript levels that depended on the presence of the functional Topo I and RecA (Figure 3B). Based on the quadrants in Figure 3B, this group could be divided into the genes whose induction required RecA and Topo I, including several members of the SOS regulon [35,36], and the genes whose repression depended on the presence of RecA and Topo I. The latter subgroup included genes encoding membrane and cell surface proteins. Thus it appeared that at least some genes in the genome were subject to the dual regulation mediated by RecA and Topo I. Since our linear model is based on the conservative assumption of a binary response that ignores any other information associated with the temporal profile of induction/repression, we refined our analysis of the *recA*–*topA* association by carefully examining the temporal profiles. Using the triplicate measurements of transcript abundances obtained after 0, 5, and 10 min of the drug treatment, we determined the initial slopes of transcriptional changes for every measurable transcript in the wild-type and *recA*− strains. First, based on the estimations of false-discovery rates [37], we identified 135 differentially induced and 210 repressed genes in either strain (*q* < 0.01). We then compared the slopes of induction or repression of the genes in the selected group. We found that the initial kinetics of induction of 32 genes and repression of 83 genes was significantly distinct between the two strains (*p* < 0.05, following the permutation test of residuals). Further comparisons between the wild-type and Δ*topA* strain (Table 2) allowed us to select 11 induced and 54 repressed genes as dependent on both *topA* and *recA*. In this second comparison, we were able to exclude the *topA*-independent regulation to examine the *topA*–*recA* association. The quantitative analysis of several randomly chosen transcripts from this set by RT-PCR confirmed the microarray results (Figure S3).

**Figure 5:**
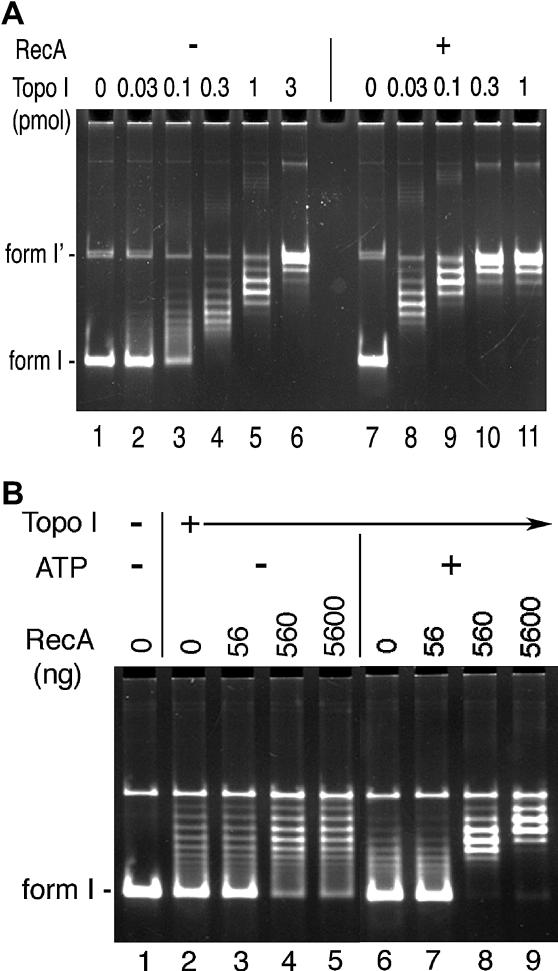
Effect of RecA on the Topo I-Catalyzed Relaxation Reaction (A) DNA relaxation reaction catalyzed by Topo I in the presence of RecA. The substrate (lanes 1 and 7), cccDNA of pBR322, was incubated with increasing amounts of Topo I in the absence (lanes: 2–6) or presence of 5,600 ng (3 nt per RecA monomer) of RecA (lanes: 8–11) at 37 °C for 30 min and resolved on the vertical agarose gel as described in Materials and Methods. (B) ATP-dependence of the RecA effect. The substrate plasmid DNA (lane: 1) was incubated with 0.1 pmol of Topo I and the indicated amounts of RecA in the absence (lanes: 2–5) or presence of ATP (lanes: 6–9).

The apparent *recA* dependence of the transcriptional responses driven by DNA relaxation prompted us to further investigate the effect(s) of RecA on the Topo I–catalyzed relaxation reaction. We observed that the RecA protein stimulated the Topo I–catalyzed relaxation of plasmid DNA in vitro by about 10-fold (Figure 5A), and that the stimulation was ATP dependent (Figure 5B). Moreover, this stimulation was enzyme specific: the E. coli RecA did not affect the relaxation reaction catalyzed by either E. coli Topo I or Staphylococcus aureus Topo I (A. Reckinger, K. S. Jeong, A. B. Khodursky, and H. Hiasa, unpublished data).

## Discussion

The average conformation of DNA inside the E. coli cell is a double helix underwound by about 6% [38,39]. Such conformation serves at least a dual purpose: it makes DNA more compact [40] and promotes reactions that use DNA as a template, including transcription [41–43]. DNA is maintained in the underwound state in large part due to the activity of DNA gyrase [44,45]. Recent studies revealed that inhibition of gyrase affects transcription of a broad spectrum of genes across the genome [5] and causes degradation of the spatial patterns of transcriptional activity in the E. coli chromosome [14]. The interpretation of the transcriptional effects of gyrase inhibition experiments is, however, complicated by the fact that the gyrase activity is required for various reactions and processes, including initiation and elongation of DNA replication [1,46], and inhibition of the gyrase activity results in DNA damage [3]. Moreover, dissecting out the relaxation-dependent effects of the gyrase inhibition cannot be done without evaluating the contribution of a relaxation activity. Discounting those effects may lead to an inadequate representation of the transcriptional consequences of gyrase inhibition and/or obscure the contribution of different regulatory processes to the transcriptional state of the cell. The goal of the proposed approach was to define which part of the response depends on replication, relaxation, DNA damage, or a combination of the effects. Since in the wild-type cells these effects are concomitant, the proper way to model their contribution to the observed transcriptional response is by fitting them simultaneously in a multivariate context. Unlike in a conventional multivariate analysis, the effects of interest could not be measured in parallel in one treatment. Instead, we had to collect measurements of the individual effects following the inhibition of gyrase in the individual mutant strains in which a specific effect of interest could not be generated, and then fit the observed changes in transcription into a global model to estimate the contributions of the individual effects.

In this report, we delineated the contribution of the effects of relaxation by Topo I, replication, and DNA damage to the transcriptional patterns of gyrase inhibition. We concluded that: (1) DNA replication is required for the formation of the spatial patterns of transcriptional activity; (2) the transcriptional response to the gyrase inhibition is coordinated between at least two modules involved in DNA maintenance, relaxation and damage response; (3) the genes whose transcriptional response to gyrase inhibition does not depend on the *topA* activity (29% of all gyrase-dependent genes) can be classified on the basis of the G + C excess in their upstream and coding sequences into, respectively, those activated and repressed by gyrase inhibition; and (4) relaxation by Topo I dominates the transcriptional response, followed by the effects of replication and RecA.

Inhibition of gyrase causes DNA relaxation. Relaxation of plasmid DNA in vivo can be carried out by Topo I [47] and Topo IV [8]; although Topo III can catalyze plasmid relaxation in vitro [48], there is no evidence for such an activity in vivo. The inhibition of gyrase by norfloxacin in the absence of the *topA* gene did not affect the distribution of topoisomers of the plasmid pBR322, indicating that under the conditions employed in this study, Topo I is the major relaxing activity in the cell. In the absence of *topA,* a fraction of plasmid DNA became hyper-negatively supercoiled ([49]; this study), indicating that the supercoiling activity of the cell is not balanced by the relaxation activity. Assuming that chromosomal DNA is also in a hyper-underwound state, such unbalancing results in somewhat limited changes in the transcriptional activity of the chromosome, including, but not limited to, activation of the *leu* cluster genes *(leuABCD* and *leuO)* and supercoiling-dependent genes *(lamB*, *proVWX,* and *malE),* and repression of genes involved in flagellar assembly and bacterial chemotaxis (ten genes). Although it is possible that the *topA* loss is compensated by the activity of another topoisomerase(s) in the cell, this compensatory effect is likely to be limited because of the unmitigated induction of supercoiling-dependent promoters such as the promoter of the *leu* cluster. Moreover, about 71% of all transcriptional changes observed as a result of the gyrase targeting were dependent on the presence of the functional *topA* gene. In fact, the observed transcriptional effects can be classified with respect to the Topo I activity as follows. Class I: the genes affected in the absence of *topA,* independent of the gyrase inactivation; class II: the genes affected in the presence of the functional *topA* when gyrase was inactivated; and class III: the genes affected in the absence of the *topA,* dependent on the gyrase inactivation. (The detailed description of classes can be found in Table S4.)

Similarly we can classify the genes whose transcriptional regulation was gyrase-dependent: class I: affected by the gyrase inhibition in both the *topA* ^–^ and *topA* ^+^ strains; class II: affected by the gyrase inhibition only in the *topA* ^+^ strain; and class III: affected by the functional gyrase in the absence of the *topA*.

Although the classes II of the two classifications are identical, class III of the gyrase-dependent classification includes genes whose transcription is sensitive to DNA (hyper)-underwinding.

The *topA*-independent changes in transcription resulting from the gyrase targeting (class I of the gyrase-dependent classification) can be explained by either invoking a residual relaxation activity or by assuming a more direct, local involvement of gyrase in a transcriptional control of the target genes. The set of such targets includes the *gyrA* and *gyrB* genes themselves (this study, [13]). Although it is plausible that another topoisomerase relaxes DNA in the vicinity of the promoters of those and other genes, the parameters of that relaxation reaction have to be comparable to the ones of the reaction catalyzed by Topo I, because the rate and the extent of activation of those genes following the gyrase inhibition was the same with or without Topo I. The stratification of the supercoiling-sensitive genes into the *topA* dependent and independent allowed us to extract the salient properties of the classified gene sets. We found that the genes repressed by the gyrase inhibition in the *topA*-independent manner had an unusually high GC content in the 5′ regions of their coding sequences. It is plausible that transcription through such regions may require facilitation by means of an efficient unwinding ahead of the moving RNA polymerase, analogous to the replication swivel [50]. The genes whose transcription was induced by the inhibition of the supercoiling activity (a somewhat enigmatic property of the supercoiling–relaxation homeostasis in the cell) in the *topA*-independent fashion can be characterized as a group on the basis of a high GC content in their upstream regions. On the basis of the length and compositional characteristics of those GC regions (see Results section), it is tempting to speculate that gyrase preferentially binds to those sites and maintains supercoiling density in their vicinity. Without the gyrase activity, the supercoiling density of DNA in those regions may drop, after the passage of a replication fork or diffusion of positive supercoils generated by transcription of the neighboring genes, leading to activation of the relaxation-inducible promoters in the region. It is also possible that the feedback up-regulation of the *topA*-independent promoters occurs through the direct de-repression in the absence of gyrase, which might normally inhibit transcription of the genes located downstream of its preferential binding sites.

The estimation of the contribution of replication to the transcriptional response elicited by the gyrase inhibition revealed, according to our model, that replication is the second factor, after relaxation by Topo I, modulating transcription globally: in the absence of replication, the response to the gyrase inhibition is subdued across the board by about 84%–89%. Sassanfar and Roberts demonstrated that the LexA cleavage occurs at the normal rate and level without DNA replication, in the presence of a quinolone drug [51]. Our finding is consistent with the fact that the formation of the damage that generates the SOS signal and presumably prevents subsequent colony formation does not depend on DNA replication. While this manuscript was in preparation, Zhao et al. reported that the replication arrest in the *dnaB*(Ts) mutant did not rescue the cells from the bactericidal effect of the quinolones [7], which may be consistent with the phenomenon observed in the present study. It is conceivable that in the absence of DNA replication, the LexA cleavage does not result in effective de-repression of the SOS regulon, which in turn may make the cells more susceptible to the drug, as was observed in the present study. The bactericidal effect of the drug can be completely reversed by an inhibitor of the transcription initiation, rifampicin, suggesting that the effect of norfloxacin in the absence of replication is mediated by transcription (unpublished data). This, along with the observations that, at the restrictive temperature, the *dnaC* mutant incorporated tritiated uracil and induced the *lac* operon by IPTG to the expected level (unpublished data, [52]), suggests that the cells do not lose their capacity to transcribe DNA in the absence of replication. That, in turn, implies that the replication itself, or the state of the cell associated with normal replication, is necessary for induction of the SOS transcriptional response and for the formation of the spatial transcriptional domains.

Spatial transcriptional domains have been observed in the chromosomes of various organisms [15,53–55]. Such spatial correlations of transcriptional activity have been attributed to the phenomenon of coordinated opening up of chromosomal regions, making the DNA template accessible to the transcriptional machinery [56]. A favorable state of the DNA template, such as, for example, within a supercoiled domain [53], can potentially be formed in parallel in multiple regions on the chromosome, giving rise to a longer-range correlation components. Despite a strong evidence for the existence of the transcriptional domains in different organisms, the mechanism(s) of the domain formation have not been addressed. Our earlier results indicated that the inhibition of DNA gyrase resulted in the diminishing of the spatial transcriptional patterns in the E. coli chromosome [14]. In corroboration of that observation, here we presented the evidence that the formation of the spatial domains in the region of the chromosome surrounding the replication terminus is particularly sensitive to the gyrase activity. Moreover, the results presented here strongly suggest that replication is required for the formation of the domains. It is plausible that coordinated transcription within, and across, domains is modulated most effectively on the newly replicated, hemi-methylated and supercoiled, DNA emerging from the replisome. Even in an asynchronous population of bacterial cells, the existence of the average replication gradient may be sufficient to detect the domain formation in newly replicated DNA. It remains to be seen to what extent replication determines the wide-range positional transcriptional effects in Streptomyces coelicolor [55] and the local effects in *S. typhimurium* [54].

The capacity to respond to DNA damage was the third factor that we included as a part of the model describing the transcriptional effects of the gyrase inhibition. The induction of the DNA damage response in E. coli depends on the *recA* and *lexA* gene products, with RecA being a positive and LexA a negative regulator of the module. In the absence of the *recA* gene, the cells are expected to become susceptible to suboptimal concentrations of the drug, likely due to their inability to mount the SOS response and repair low-level DNA damage induced by the quinolones [3]. Our results were consistent with that expectation. Additionally, it appears that the inability to cope with DNA damage, or at least a quinolone-induced type of damage, is accompanied by a more generic stress response, as manifested by up-regulation of genes encoding folding and ushering proteins *(dnaJ*, *dnaK*, *htpG,* and *yrfI)*. More surprising, however, was our finding that transcription of the dozens of genes was affected by both *recA* and *topA*. Although uncovered initially on purely statistical grounds by modeling the parameters obtained in different mutant strains, this interaction appears to be real and is manifested by the altered initial rates of repression, or activation, of transcription as well as, in some cases, the end-point levels of the transcripts following inhibition of the supercoiling activity in the Δ*recA* strain. Although such an outcome could have been brought about either indirectly, by the state of the DNA template in the presence of persistent damage, or directly, through the interference of RecA with the relaxation reaction, we were intrigued by the cases of accelerated repression in the *recA* ^+^ background. Most prominently this was exemplified by the repression of transcription of the *ompF* gene. Since transcription of the gene is known to be dependent on negative supercoiling and inhibited by DNA relaxation [57], one would have to assume that the functional RecA, either directly or indirectly, accelerates this process. We examined the possibility of a direct effect by carrying out the Topo I relaxation assay in vitro with or without RecA and found that, at the physiological concentrations, the RecA protein stimulated the Topo I catalyzed relaxation reaction by about 10-fold in the ATP-dependent manner. Although it is possible that the stimulation is the result of the conformational changes induced in the supercoiled plasmid DNA by the RecA protein, making it a better substrate for the Topo I reaction, the species-dependent enzyme specificity suggests that the stimulation cannot be explained solely on the basis of the induced preferential conformation of the substrate. The biochemical mechanism of the interaction between Topo I and RecA is currently under investigation. The interaction on the level of transcription may help explain the observation made by E. Witkin and co-workers more than 20 y ago where they showed that the efficient shut-down of the OmpF synthesis in the cells treated with nalidixic acid required the functional RecA protein [58]. It is worth noting that the synthetic conditional interactions between the mutant *recA* and *topA* alleles affecting cell physiology and viability have been reported before [59–62] but, to the best of our knowledge, this is the first study that implicated the two activities in the concerted regulation of transcription and the direct interaction.

Genome-wide transcriptional profiles often result from contributions of multiple effects including, but not limited to, transcript initiation and decay [63], differentially controlled regulatory networks, and indirect effects on a transcriptional state of cells. Assuming that these effects can be separated, genetically or by other means, a compound transcriptional profile can be explained in terms of contributing effects, thus allowing for a more realistic, accurate, and meaningful representation of the transcriptional activity of a genome. Here we demonstrated the utility of such an approach by delineating the transcriptional response of the bacterial cells to the gyrase inhibition. The multivariate approach allowed us to interpret the complex profile as a function of three effects known to be triggered in the cells by the loss of the gyrase function. The analysis not only yielded all of the expected features of the response, but also uncovered subtleties of the individual effects and the interactions between them, which are readily missed by a conventional pair-wise analysis. Moreover, the response can be fully explained by the combination of the three factors: DNA relaxation by Topo I, DNA damage, and inhibition of DNA replication.

## Materials and Methods

### Strains, cultures, and treatments.

A Δ*recA* mutation in an E. coli K-12 strain MG1655 was constructed by a Red recombinase–mediated replacement [64]. A strain with Δ*topA* [21] was obtained from the E. coli Genetic Stock Center, and the Δ*topA* allele was transferred into MG1655 by P1 transduction. To assess DNA replication effects, a temperature-sensitive *dnaC* allele linked to the Tn10 was transferred into the MG1655 strain. Bacteria were cultured in Luria-Bertani (LB) or Vogel-Bonner (VB) minimal medium supplemented with 0.2% glucose [65], and the analysis is reported for the wild-type and mutant strains grown and treated in the minimal medium. Bacterial cultures were grown in shake flasks with standard aeration to an optical density (measured at OD_600_) of approximately 0.4, at which point norfloxacin (1 μg/ml) was added, and samples were collected at 5, 10, 15, and 20 min after the addition. The norfloxacin treatment of a *dnaC*(Ts) mutant was done when the culture reached the same optical density, but after the initiation of DNA replication was blocked at a non-permissive temperature for 90 min [66].

### Microarray/RT-PCR.

Relative mRNA abundances between treated and non-treated cells, or a mutant and the wild type, were compared using E. coli whole-genome DNA microarrays containing 99% of all annotated open reading frames and the stable RNA genes. The protocols for slide preparation, RNA purification, reverse transcription with the Cy-dyes, hybridization, and image scanning were described before [67]. Briefly, total RNA purified using hot phenol extraction was subjected to reverse transcription with a Cy3 or a Cy5 fluorophore (Amersham, Little Chalfont, United Kingdom). The fluorescent probes were hybridized to an array at 65 °C for 6 h. Intensities in both channels were smoothed using the lowess method [68], and dye- and array-specific noise was removed using the analysis of variance (ANOVA) error model [69]. A detailed description of statistical analysis of DNA microarray data is provided in the supporting information Text S1. In pair-wise comparisons, differentially expressed genes were identified at an estimated false discovery rate of less than 1% using the SAM package [37]. Real-time PCR for RNA quantification was carried out using Syber-Green kit and ABI prism 7900 (Applied Biosystems, Foster City, California, United States) according to the manufacturer's protocol.

### Linear model analysis.

To characterize the effects of inhibition of gyrase by the drug in different genetic backgrounds, a linear regression model was fitted for each gene as follows:

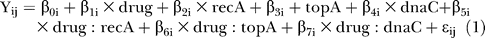
where Y***_ ij_*** represents the log*_2_* intensity of gene *i* (*i* = 1,2,3,…4,069) and channel *j* (*j* = 1,2,…32). The coding information of the variables and the design matrix can be found in Text S1. For the sake of simplicity, we will omit the subscript *i* from the following presentation. In this model, β_0_ represents the gene expression level for a wild-type strain without drug treatment; β_1_ is the coefficient for the drug effect on the wild type; the β_2_ *,* β_3_ ***,*** and β_4_ correspond to transcriptional differences in the untreated Δ*recA,* Δ*topA,* and *dnaC*(Ts) strains, respectively, relative to the untreated wild-type sample; the coefficients β_5_ *,* β_6_ *,* and β_7_ represent the difference in treatment effects between wild-type and mutant strains (Δ*recA,* Δ*topA,* and *dnaC*(Ts)*,* respectively). *ɛ* is the measurement error that stands for the difference between the observed and the model-predicted response, and *ɛ* is assumed to follow an independent normal distribution. The point and variance estimation of the coefficients were performed in R package (http://www.r-project.org) and the corresponding *p*-values were used to do the inference. (See Text S1 and Table S3 for details).

### DNA relaxation assays.

Reaction mixtures contained 50 mM Tris-HCl (pH 8.0), 100 mM potassium glutamate, 10 mM MgCl_2_, 5 mM ATP (or the indicated cofactor), 100-mg/ml BSA, 10% (w/w) glycerol, 0.29 μg [ = 100 fmol] of pBR322 form I DNA, either 0 or 5,600 ng [ = 3 bp per RecA monomer] (or the indicated amounts) of RecA, and 0 or 0.1 pmol (or the indicated amounts) of Topo I. The reaction mixtures were assembled on ice. First, RecA was added and incubated at 37 °C for 5 min, then Topo I was added and incubated at 37 °C for 30 min. Reactions were terminated by the addition of EDTA to 25 mM and incubation at 37 °C for 2 min. SDS and proteinase K were then added to 1% and 100 mg/ml, respectively, and the incubation was continued for an additional 15 min. The DNA products were purified by extraction of the reaction mixtures with phenol-chloroform (1:1, v/v) and then analyzed by electrophoresis through vertical 1.2% agarose gels (14 × 10 × 0.3 cm) at 2 V/cm for 12 h in a running buffer of 50 mM Tris-HCl (pH 7.9), 40 mM sodium acetate, and 1 mM EDTA (TAE buffer). Gels were stained with ethidium bromide and photographed using an Eagle Eye II system (Stratagene, La Jolla, California, United States).

To assess DNA supercoiling in vivo, the plasmid pBR322 was transformed into the wild-type MG1655 and the *topA* deletion mutant. Cells in a mid-exponential phase were treated with 1-, 10-, and 50-μg/ml norfloxacin for 10 min, and the plasmid DNA was isolated with Midipreps kit (Promega, Madison, Wisconsin, United States). Topoisomers were resolved in a 1% agarose gel in TAE buffer supplemented with 3-μg/ml chloroquine, at 3 V/cm for 16–20 h.

## Supporting Information

Figure S1 Distribution of Topoisomers of the Plasmid pBR322 Isolated from the Wild-Type Cells or a *topA* Mutant after the Norfloxacin Treatment (A) Cells were treated with indicated concentrations of norfloxacin, and the plasmid DNA was isolated after 10 min of treatment.
(B) Cells were treated with norfloxacin after the temperature downshift from 37 °C to 30 °C.
(1.1 MB EPS)

Figure S2 Effect of Replication Initiation Arrest on Transcriptional Activity of the SOS Regulon Transcript levels, as measured by DNA microarrays, of representative genes of the SOS regulon were compared between the wild type ([A] upper panel) and the *dnaC* mutant ([B] lower panel). Although the norfloxacin-treated wild-type cells exhibited significant induction of the SOS genes in 5 min, the *dnaC* mutant cells showed a relatively low level of induction in the absence of ongoing DNA replication.
(949 KB EPS)

Figure S3 Quantitative Analysis of Relative Transcript Levels by RT-PCR (A) The recA-dependent transcript levels of the supercoiling-dependent genes were determined at 0, 5, and 10 min of the norfloxacin treatment.
(B) Correspondence between the transcript levels estimated from microarray and RT-PCR measurements. The mRNA abundances at 5 min after the norfloxacin treatment were compared with mRNA abundances of the non-treated samples in three independent biological replicates. The RT-PCR measurements were done three times in one randomly chosen sample. Error bars represent two standard errors of the mean.
(1.4 MB EPS)

Table S1 Functional Classification of the Differentially Expressed Genes (88 KB DOC)

Table S2 Cell Viability during the Norfloxacin Treatment (28 KB DOC)

Table S3 Coefficients and *p*-Values Determined by the Linear Model (751 KB XLS)

Table S4 Transcriptional Effects with Respect to the Topo I Activity (31 KB DOC)

Text S1 Overall Analysis Plan and the Design Matrix for the Linear Model (88 KB DOC)

### Accession Numbers

The SwissProt (http://www.expasy.org/sprot) accession numbers for genes described in this study are *acnB* (P36683)*, aldA* (P25553)*, argR* (P15282)*, argS* (P11875)*, cydD* (P29018)*, dnaC* (P07905)*, dnaJ* (P08622)*, dnaK* (P04475)*, fadL* (P10384)*, fruR* (P21168)*, fumC* (P05042)*, gltA* (P00891)*, glyA* (P00477)*, gor* (P06715)*, htpG* (P10413)*, lamB* (P02943)*, leuA* (P09151)*, leuB* (P30125)*, leuC* (P30127)*, leuD* (P30126)*, leuO* (P10151)*, lexA* (P03033)*, lldD* (P33232)*, lldP* (P33232)*, malE* (P02928)*, metK* (P04384)*, ompF* (P02931)*, parC* (P20082)*, parE* (P20083)*, pdhR* (P06957)*, pps* (P23538)*, proV* (P14175)*, proW* (P14176)*, proX* (P14177), *rbsD* (P04982)*, recA* (P03017)*, rfaZ* (P27241)*, speB* (P16936)*, sulA* (P08846)*, tolC* (P02930), *topA* (P06612)*, topB* (P14294)*, trxA* (P00274)*, yccS* (P75870)*, ychB* (P24209), *yecD* (P37347)*,* and *yrfI* (P45803).

The Gene Expression Omnibus (http://www.ncbi.nlm.nih.gov/projects/geo) accession number for the transcript levels of the four strains used in this study is GSE 4408.

## Abbreviations

bp: base pair
kb: kilobase
SOM: self-organizing maps
Topo I: topoisomerase I
