# Supplementary material for: Analysis of Pleiotropic Transcriptional Profiles: A Case Study of DNA Gyrase Inhibition
Source: PLoS Genet. 2006 Sep 29;2(9):e152. doi: 10.1371/journal.pgen.0020152 (PMC1584274; doi:10.1371/journal.pgen.0020152)
Supplement: Table S1 — (88 KB DOC) [file pgen.0020152.st001.doc]

**Table S1.** Functional Classification of Differentially Expressed Genes

| **Strain** | **Number  of Genes** | **Functional Categories** | **Exp*** | **Obs** | ***p-* value**^§^ |
| --- | --- | --- | --- | --- | --- |
| Wild type | Up-regulation (269 genes) | DNA synthesis, Modification, Degradation | 73 | 21 | 3.7E-09 |
|  |  | Global Regulatory Functions, Adaptation | 78 | 15 | 1.3E-04 |
|  |  | Central Intermediary Metabolism | 290 | 30 | 8.1E-03 |
|  |  | Biosynthesis of Co-factors | 67 | 10 | 1.2E-02 |
|  |  | Nucleotide Biosynthesis | 22 | 5 | 1.3E-02 |
|  |  | Cell Division, Protection Responses | 57 | 7 | 7.9E-02 |
|  |  | Protein synthesis, Modification, Degradation, Ribosome Constituent | 59 | 6 | 1.9E-01 |
|  |  | Transport / binding Proteins | 239 | 13 | 8.1E-01 |
|  | Down-regulation (344 genes) | Amino acids Biosynthesis | 68 | 26 | 8.8E-12 |
|  |  | Nucleotide Biosynthesis | 22 | 9 | 3.7E-05 |
|  |  | Biosynthesis of Co-factors | 67 | 14 | 1.2E-03 |
|  |  | Transport / binding Proteins | 239 | 31 | 9.8E-03 |
|  |  | Global Regulatory Functions, Adaptation | 78 | 11 | 6.3E-02 |
|  |  | DNA synthesis, Modification, Degradation | 73 | 10 | 8.6E-02 |
|  |  | Protein synthesis, Modification, Degradation, Ribosome Constituent | 59 | 7 | 2.3E-01 |
|  |  | Cell Envelop | 66 | 7 | 3.2E-01 |
|  |  | Central Intermediary Metabolism | 290 | 26 | 4.1E-01 |
| *recA*  mutant | Up-regulation (313 genes) | Global Regulatory Functions, Adaptation | 78 | 21 | 2.0E-07 |
|  |  | DNA synthesis, Modification, Degradation | 73 | 13 | 3.2E-03 |
|  |  | Folding and Ushering Proteins | 49 | 8 | 3.1E-02 |
|  |  | Cell Division, Protection Responses | 57 | 8 | 6.8E-02 |
|  |  | Central Intermediary Metabolism | 290 | 29 | 8.1E-02 |
|  |  | Biosynthesis of Co-factors | 67 | 7 | 2.5E-01 |
|  |  | Transport / binding Proteins | 239 | 13 | 9.4E-01 |
|  | Down-regulation (351 genes) | Amino acids Biosynthesis | 68 | 22 | 2.0E-08 |
|  |  | Transport / binding Proteins | 239 | 35 | 1.0E-03 |
|  |  | Nucleotide Biosynthesis | 22 | 6 | 8.9E-03 |
|  |  | Biosynthesis of Co-factors | 67 | 12 | 1.1E-02 |
|  |  | Cell Envelop | 66 | 10 | 5.4E-02 |
|  |  | Central Intermediary Metabolism | 290 | 32 | 8.2E-02 |
|  |  | Global Regulatory Functions, Adaptation | 78 | 9 | 2.3E-01 |
|  |  | DNA synthesis, Modification, Degradation | 73 | 6 | 6.1E-01 |
| *topA*  mutant | Up-regulation (241 genes) | DNA synthesis, Modification, Degradation | 73 | 18 | 1.2E-07 |
|  |  | RNA synthesis, Modification, Degradation | 18 | 5 | 3.2E-03 |
|  |  | Global Regulatory Functions, Adaptation | 78 | 11 | 5.7E-03 |
|  |  | Cell Division, Protection Responses | 57 | 7 | 4.9E-02 |
|  |  | Central Intermediary Metabolism | 290 | 24 | 5.6E-02 |
|  |  | Folding and Ushering Proteins | 49 | 5 | 1.6E-01 |
|  |  | Amino acids Biosynthesis | 68 | 6 | 2.1E-01 |
|  |  | Protein synthesis, Modification, Degradation, Ribosome Constituent | 59 | 5 | 2.7E-01 |
|  |  | Biosynthesis of Co-factors | 67 | 5 | 3.6E-01 |
|  |  | Transport / binding Proteins | 239 | 7 | 9.9E-01 |
|  | Down-regulation (243 genes) | Biosynthesis of Co-factors | 67 | 12 | 4.9E-04 |
|  |  | Amino acids Biosynthesis | 68 | 12 | 5.7E-04 |
|  |  | Central Intermediary Metabolism | 290 | 23 | 9.4E-02 |
|  |  | Protein synthesis, Modification, Degradation, Ribosome Constituent | 59 | 5 | 2.8E-01 |
|  |  | Global Regulatory Functions, Adaptation | 78 | 6 | 3.2E-01 |
|  |  | Transport / binding Proteins | 239 | 16 | 3.5E-01 |
|  |  | Cell Envelop | 66 | 5 | 3.6E-01 |
|  |  | DNA synthesis, Modification, Degradation | 73 | 5 | 4.4E-01 |
| *dnaC*  mutant | Up-regulation (24 genes) | DNA synthesis, Modification, Degradation | 73 | 5 | 5.3E-05 |
|  | Down-regulation (52 genes) | Cell Envelop | 66 | 4 | 9.5E-03 |

^§^To assess the significance of a functional enrichment of groups of genes, the *p*-values were determined using the statistical test based on

the hypergeometric distribution. The categories with *p*<0.05 or at least 5 genes are shown.

*The expected number of genes and the functional classification are based on [1].

**References**

1. Carbone, A., Zinovyev, A., Kepes, F. (2003) Codon adaptation index as a measure of dominating codon bias. Bioinformatics 19: 2005-2015.
