# Supplementary material for: Analysis of Pleiotropic Transcriptional Profiles: A Case Study of DNA Gyrase Inhibition
Source: PLoS Genet. 2006 Sep 29;2(9):e152. doi: 10.1371/journal.pgen.0020152 (PMC1584274; doi:10.1371/journal.pgen.0020152)
Supplement: Table S2 — (28 KB DOC) [file pgen.0020152.st002.doc]

**Table S2**. Cell Viability during the Norfloxacin Treatment

| Treatment time (min) | 0 | 5 | 10 | 15 | 20 |
| --- | --- | --- | --- | --- | --- |
| Wild type | 1 | 0.80 (± 0.09)^§^ | 0.52 (± 0.08) | 0.38 (± 0.11) | 0.25 (± 0.03) |
| *recA-* | 1 | 0.30 (± 0.15) | 0.21 (± 0.07) | 0.13 (± 0.00) | 0.02 (± 0.02) |
| *topA-* | 1 | 0.88 (± 0.05) | 0.84 (± 0.11) | 0.78 (± 0.12) | 0.63 (± 0.09) |
| *dnaC*(Ts)* | 1 | 0.50 (± 0.04) | 0.35 (± 0.08) | 0.28 (± 0.08) | 0.12 (± 0.10) |

^§^Values in parenthesis correspond to standard deviations obtained on 3 independent cultures.

*The viability of *dnaC*(Ts) cells was measured after 90 min at the nonpermissive

temperature to inhibit initiation of DNA replication.
