# Supplementary material for: Analysis of Pleiotropic Transcriptional Profiles: A Case Study of DNA Gyrase Inhibition
Source: PLoS Genet. 2006 Sep 29;2(9):e152. doi: 10.1371/journal.pgen.0020152 (PMC1584274; doi:10.1371/journal.pgen.0020152)
Supplement: Table S4 — (31 KB DOC) [file pgen.0020152.st004.doc]

**Table S4.** Transcriptional Effects with Respect to the Topo I Activity

(Top 5 most enriched for functional categories with gene samples are listed.)

**Class I:**

**- 132 activated genes only in *topA-***

Central Intermediary Metabolism : *ackA, cysH, cysJ, dniR, gcvA, metK, pfkB, pta, pyrG, rfbA*

Cell Division, Protection Responses: *gidA, gidB, katE, mioC, mopB*

Amino acids Biosynthesis: *argC, argE, argH, aroF, metJ*

Folding and Ushering Proteins: *hslV, infA, slyD, tufB*

Transport / binding Proteins: *artP, fadL, rbsD*

**- 117 repressed genes only in *topA-*:**

Central Intermediary Metabolism: *aceB, aceE, aceK, dadA, dld, fadB, gcbR, mdh, pgi, pykF, uxuA*

Transport / binding Proteins: *aroP, modF, mtlA, nhaB, pstS, ydhP*

Biosynthesis of Co-factors: *bioB, moaA, nadC, panD, pncB*

Global Regulatory Functions, Adaptation: *csrA, glgB, imp, uspA*

Amino acids Biosynthesis: *asd, dapA, ilvC*

**Class II:**

**- 160 activated genes in wild type, but not in *topA-*:**

Central Intermediary Metabolism : *deoA, deoB, deoC, icdA, ldhA, nrdA, nrdB, ntpA, pckA, pldB, ppsA, rpiA, sdaA, tktA, trxB, udp, upp, uxuR*

Transport / binding Proteins : *brnQ, cydD, cysZ, glnH, lysP, nhaA, oppB, proP, ybeJ*

Biosynthesis of Co-factors: *bioA, gor, hemL, moeA, ribA, trxA, ubiG*

Global Regulatory Functions, Adaptation: *lpxC, osmC, phoB, rfaQ, rfaZ, sspA*

DNA synthesis, Modification, Degradation: *dinG, hrpA, sbcD, sbmC, ssb*

**- 211 repressed genes in wild type, but not in *topA-*:**

Transport / binding Proteins: *artJ, btuE, dppB, fepA, fepB, fes, fhuE, hisJ, kgtP, livJ, malF, malK, manX,* *manY, mglB, potA, potF, proV, ptsH, tonB, ugpB, yifK*

Amino acids Biosynthesis: *alr, argA, argD, argF, argG, argI, aspC, cbl, cysM, dapB, glnL, hisG, metA, metE, metH, trpE*

Central Intermediary Metabolism: *adhE, atpG, gatR_2, glnK, gltB, gsk, malM, malP, metF, mtlD, nuoN, ppc, sfcA, speE, zwf*

Global Regulatory Functions, Adaptation: *bfr, cspA, cytR, mdoB, msbB, nlpA, nlpC, pssA, pssR*

Nucleotide Biosynthesis: *adk, apbA, carA, guaA, guaC, ndk, pyrD, pyrF*

**Class III:**

**- 84 activated genes in wild type and *topA-*:**

Central Intermediary Metabolism: *add, deoD, fbaB, fbp, galE, gntR, kbl, nagB, pflA, speB*

DNA synthesis, Modification, Degradation: *dfp, dnaG,, dnaT, gyrA, gyrB, holD, nfo, rph, umuD*

Global Regulatory Functions, Adaptation: *fnr, fur, hslJ, lgt, oxyR, psd, rpoD*

RNA synthesis, Modification, Degradation: *greA, heaP, yjjT*

Protein synthesis, Modification, Degradation, Ribosome Constituent: *gltX, metG, trmA*

**- 95 repressed genes in wild type and *topA-*:**

Central Intermediary Metabolism: *acnB, aldA, cybB, cysD, gcvP, gluM, gpmA, malT, qor*

Amino acids Biosynthesis: *aroG, gdhA, ilvB, ilvD, ilvG_1, lysC, serA*

Transport / binding Proteins: *argT, dctA, dppA, livK, pstB, putP, uhpA*

Biosynthesis of Co-factors: *hemN, nadA, nrdH, pdxK*

Protein synthesis, Modification, Degradation, Ribosome Constituent: *alaS, hlpA, hns, hupA*
