# Supplementary material for: Analysis of Pleiotropic Transcriptional Profiles: A Case Study of DNA Gyrase Inhibition
Source: PLoS Genet. 2006 Sep 29;2(9):e152. doi: 10.1371/journal.pgen.0020152 (PMC1584274; doi:10.1371/journal.pgen.0020152)
Supplement: Text S1 — (88 KB DOC) [file pgen.0020152.sd001.doc]

**Supporting information Text S1.**

***Overall analysis plan***

The main goal of the analysis was to uncouple the transcriptional effects of relaxation, repair and replication processes. To achieve this goal, we first used a simple and straightforward pair-wise comparison method to evaluate the differences in transcriptional activity between strains. Table 1 and Table 2 summarize the most significant results of such comparisons. Additionally, the results of pair-wise comparisons were used to qualitatively assess the consistency between the experimental observations and the available biological knowledge. The evidence generated in pair-wise comparisons supported our assertion that the mutant strains can be used to decompose the transcriptional response in a wild type into the effects associated with different biological process. Next, we formalized a linear model as a tool to analyze this type of data. We fitted the model and used the estimated parameters for further analysis (Fig. 3), which resulted in an experimentally testable hypothesis (Fig. 5). We describe the pair-wise comparison method and linear model method in detail in the following sections. In addition to the inferential analysis and post-inferential classification, we carried a pure exploratory analysis of the data sets using Self-Organizing Feature Maps as well as the analysis of spatial patterns using temporal correlations within moving windows.

The image in Fig. 1 was generated using Matlab 6.0 (http://www.mathworks.com); the image in Fig. 2 was generated using Self-Organizing Map toolbox (http://www.cis.hut.fi/projects/somtoolbox).

***Pair-wise comparisons between bacterial strains (Table 1 and Table 2)***

First, we used SAM [1] to identify the genes that were differentially expressed between treated and non-treated bacteria. Specifically, transcript levels were measured with two-channel microarrays, in which one channel contained information about transcript abundances in a norfloxacin treated sample and another channel corresponded to an untreated reference. The treated cells, sampled at four time points (5, 10, 15 and 20 min) after the beginning of the treatment, were compared with the cells collected before the treatment (0 min). The global lowess normalization method [2] was applied to microarray data based on the scatter plot of log ratios versus their intensities. We used the MAANOVA package [3] to remove the array-specific noise in 4 arrays, which we treated as biological replicates. Following the normalization, we performed two-class SAM using 4 intensities from norfloxacin-treated samples and 4 from non-treated samples. To determine the differentially expressed genes in each strain the same criteria (FDR < 1%, at least 1.5-fold in the average log ratios) were used for all 4 strains.

Then we compared the transcriptional responses between different strains. This analysis was carried out *only* on the genes that were identified by SAM as differentially expressed in response to the drug in the wild type. To this end, we took the expression levels, measured as the log_2_ ratios in the comparisons of treated samples vs. untreated reference, of the genes differentially affected in the wild and compared those with the expression levels, similarly measured, of *the same genes* in mutant strains. The difference between the means of corresponding samples, each sample being represented by 4 time points treated as replicates, was evaluated using Student t-test and p<0.01 was chosen as a cut-off for the summary representation. Table 1 shows the results of the analysis: consistent with the biological expectation, the most reproducibly affected genes in the wild type strain are related to the SOS response. Similarly we compared the transcriptional responses between the wild type and *topA*- strain and summarized the results in Table 2. Because the effect of Topoisomerase I (Topo I, encoded by the *topA* gene) on the transcription of the supercoiling sensitive genes, SSGs, has not been investgated, we classified the differentially expressed genes in the wild type strain with respect to the Topo I activity (Table 2). To determine the significance of the overlap between the gene lists classified in the *topA*-dependent manner and the SSGs, we used statistical tests based on the hypergeometric probability distribution. Due to the lack of expectations about the transcriptional activity in the absence of ongoing replication in a *dnaC*(Ts) mutant, we evaluated microarray performance for the consistency of the observed response in different media or at different time points following the drug treatment (Figure 1 and Figure S2).

**Linear model**

Next, we demonstrated that the data, which can be characterized by a relatively low variance and biological soundness, can be analyzed within a true multivariate framework. The proposed model represents a formal, self-consistent, and adequate approach to the analysis of the data where the transcriptional response is likely to be a combination of multiple effects. Within the adopted linear model, multiple effects can be easily combined together. In the model, different time points are treated, again, as replicates and the categorical variables in the model are as follows:

Y ***_ij_*** ***= β_0i_ +β_1i_×drug + β_2i_×recA + β_3i_×topA + β_4i_×dnaC + β_5i_×drug:recA +β_6i_ ×drug:topA+ β_7i_×drug:dnaC + ε_ij_***

drug=1 when using drug (all channels with treated samples) ;

drug=0 when not using drug (all channels with non-treated samples);

recA=1 for ∆*recA* strain; recA=0 for wild type (WT), ∆*top*A or *dnaC*(Ts) strains

topA=1 for ∆*top*A strain; topA=0 for WT, ∆*rec*A or *dnaC*(Ts) strains

dnaC=1 for *dnaC*(Ts) strain; dnaC=0 for WT, ∆*rec*A or ∆*top*A strains

The design matrix for 16 two-channel microarrays (4 strains) is as follows:

|  | Drug | recA | topA | dnaC | Drug x recA | Drug x topA | Drug x dnaC |
| --- | --- | --- | --- | --- | --- | --- | --- |
| WT, Drug | 1 | 0 | 0 | 0 | 0 | 0 | 0 |
| WT, no drug | 0 | 0 | 0 | 0 | 0 | 0 | 0 |
| WT, Drug | 1 | 0 | 0 | 0 | 0 | 0 | 0 |
| WT, no drug | 0 | 0 | 0 | 0 | 0 | 0 | 0 |
| WT, Drug | 1 | 0 | 0 | 0 | 0 | 0 | 0 |
| WT, no drug | 0 | 0 | 0 | 0 | 0 | 0 | 0 |
| WT, Drug | 1 | 0 | 0 | 0 | 0 | 0 | 0 |
| WT, no drug | 0 | 0 | 0 | 0 | 0 | 0 | 0 |
| recA, Drug | 1 | 1 | 0 | 0 | 1 | 0 | 0 |
| recA, no drug | 0 | 1 | 0 | 0 | 0 | 0 | 0 |
| recA, Drug | 1 | 1 | 0 | 0 | 1 | 0 | 0 |
| recA, no drug | 0 | 1 | 0 | 0 | 0 | 0 | 0 |
| recA, Drug | 1 | 1 | 0 | 0 | 1 | 0 | 0 |
| recA, no drug | 0 | 1 | 0 | 0 | 0 | 0 | 0 |
| recA, Drug | 1 | 1 | 0 | 0 | 1 | 0 | 0 |
| recA, no drug | 0 | 1 | 0 | 0 | 0 | 0 | 0 |
| topA, Drug | 1 | 0 | 1 | 0 | 0 | 1 | 0 |
| topA, no drug | 0 | 0 | 1 | 0 | 0 | 0 | 0 |
| topA, Drug | 1 | 0 | 1 | 0 | 0 | 1 | 0 |
| topA, no drug | 0 | 0 | 1 | 0 | 0 | 0 | 0 |
| topA, Drug | 1 | 0 | 1 | 0 | 0 | 1 | 0 |
| topA, no drug | 0 | 0 | 1 | 0 | 0 | 0 | 0 |
| topA, Drug | 1 | 0 | 1 | 0 | 0 | 1 | 0 |
| topA, no drug | 0 | 0 | 1 | 0 | 0 | 0 | 0 |
| dnaC, Drug | 1 | 0 | 0 | 1 | 0 | 0 | 1 |
| dnaC, no drug | 0 | 0 | 0 | 1 | 0 | 0 | 0 |
| dnaC, Drug | 1 | 0 | 0 | 1 | 0 | 0 | 1 |
| dnaC, no drug | 0 | 0 | 0 | 1 | 0 | 0 | 0 |
| dnaC, Drug | 1 | 0 | 0 | 1 | 0 | 0 | 1 |
| dnaC, no drug | 0 | 0 | 0 | 1 | 0 | 0 | 0 |
| dnaC, Drug | 1 | 0 | 0 | 1 | 0 | 0 | 1 |
| dnaC, no drug | 0 | 0 | 0 | 1 | 0 | 0 | 0 |

In this model, each coefficient has its own biological meaning, (see the main article) and, moreover, the coefficients themselves can be explored further, producing unexpected biological insights (Fig. 3). Additionally, the coefficients estimated from the model can be used directly in the inferential analysis, where they perform at least as well as the estimated ratios (data not shown).

***Comparison of initial transcriptional responses between a wild type and a recA- strain***

We observed that the initial rates of the transcriptional response of the DNA supercoiling-sensitive genes were different between a wild type strain and a *recA*- mutant. To further explore the difference in the rates of transcriptional activation and repression between the two strains, we collected 3 biological replicates at each of the early time points (0, 5, and 10 min) and estimated the corresponding transcript abundance levels using the MAANOVA model [3]. Next, the estimated transcript levels at the three time points have been fit into a line and the confidence interval for each value of the slope was determined by permuting the model’s residuals, re-fitting the line, and re-estimating the slope. The significance of the difference between the magnitudes of the slopes of the fitted lines for the wild type and *recA*- has been evaluated after 1,000 permutations. The kinetics of the transcriptional response, in the wild type and the mutant, for several randomly selected genes with estimated, from the microarray analysis, significantly different initial rates have been confirmed with RT-PCR (Figure S3).

**References**

1. Tusher VG, Tibshirani R, Chu G (2001) Significance analysis of microarrays applied to the ionizing radiation response. Proc Natl Acad Sci U S A 98: 5116-5121.

2. Yang YH, Dudoit S, Luu P, Lin DM, Peng V, et al. (2002) Normalization for cDNA microarray data: a robust composite method addressing single and multiple slide systematic variation. Nucleic Acids Res 30: e15.

3. Cui X, Churchill GA (2003) Statistical tests for differential expression in cDNA microarray experiments. Genome Biol 4: 210
